# Update of incidence and antimicrobial susceptibility trends of *Escherichia coli* and *Klebsiella pneumoniae* isolates from Chinese intra-abdominal infection patients

**DOI:** 10.1186/s12879-017-2873-z

**Published:** 2017-12-18

**Authors:** Hui Zhang, Qiwen Yang, Kang Liao, Yuxing Ni, Yunsong Yu, Bijie Hu, Ziyong Sun, Wenxiang Huang, Yong Wang, Anhua Wu, Xianju Feng, Yanping Luo, Yunzhuo Chu, Shulan Chen, Bin Cao, Jianrong Su, Qiong Duan, Shufang Zhang, Haifeng Shao, Haishen Kong, Bingdong Gui, Zhidong Hu, Robert Badal, Yingchun Xu

**Affiliations:** 1 Division of Microbiology, Peking Union Medical College Hospital, Peking Union Medical College, Chinese Academy of Medical Sciences, No. 1 Shuaifuyuan, Wangfujing Street, Beijing, 100730 China; 2 Division of Microbiology, The First Affiliated Hospital, Sun Yat-Sen University, Guangzhou, 510080 China; 3 Division of Microbiology, Ruijin Hospital, School of Medicine, Shanghai Jiaotong University, Shanghai, 200025 China; 4 Department of Infectious Diseases, SirRunRun Shaw Hospital, School of Medicine, Zhejiang University, Hangzhou, 310016 China; 5 Division of Microbiology, Zhongshan Hospital of Fudan University, Shanghai, 200032 China; 6 Department of Laboratory Medicine, Tongji Hospital, Tongji Medical College, Huazhong University of Science and Technology, Wuhan, 430030 China; 7 Division of Microbiology, The First Affiliated Hospital of Chongqing Medical University, Chongqing, 400016 China; 8 Department of Laboratory Medicine, Shandong Provincial Hospital Affiliated to Shandong University, Jinan, 250021 China; 9 Infection Control Center, Xiangya Hospital, Central South University, Changsha, 410008 China; 10 Division of Microbiology, The First Affiliated Hospital of Zhengzhou University, Zhenzhou, 450052 China; 11 Department of Microbiology, The Chinese PLA General Hospital, Beijing, 100853 China; 12 Division of Microbiology, The First Affiliated Hospital of Chinese Medical University, Shenyang, 110001 China; 13 Division of Microbiology, The First Affiliated Hospital of Harbin Medical University, Harbin, 150001 China; 14 Department of Respiratory and Crtical Care Medicine, Clinical Microbiology and Infectious Disease Laboratory, China-Japan Friendship Hospital, Beijing, 100029 China; 15 Department of Clinical Laboratory, Beijing Friendship Hospital of Capital Medical University, Beijing, 100020 China; 16 Microbiology Laboratory, Jilin Province People’s Hospital, Changchun, 130021 China; 17 Division of Microbiology, Haikou People’s Hospital, Haikou, 570208 China; 18 Division of Microbiology, General Hospital of Nanjing Military Command, Nanjing, 210002 China; 19 Department of Microbiology, The First Affiliated Hospital of Zhejiang University, Hangzhou, 310003 China; 20 Clinical laboratory, The Second Affiliated Hospital of Nanchang University, Nanchang, 330006 China; 21 Division of Microbiology, Tianjin Medical University General Hospital, Tianjing, 300052 China; 22 Division of Microbiology, International Health Management Associates, Schaumburg, IL 60173-3817 USA

**Keywords:** Carbapenems, Extended spectrum beta-lactamase, Intra-abdominal infection, *Escherichia coli*, *Klebsiella pneumoniae*

## Abstract

**Background:**

To evaluate in vitro susceptibilities of aerobic and facultative Gram-negative bacterial (GNB) isolates from intra-abdominal infections (IAIs) to 12 selected antimicrobials in Chinese hospitals from 2012 to 2014.

**Methods:**

Hospital acquired (HA) and community acquired (CA) IAIs were collected from 21 centers in 16 Chinese cities. Extended spectrum beta-lactamase (ESBL) status and antimicrobial susceptibilities were determined at a central laboratory using CLSI broth microdilution and interpretive standards.

**Results:**

From all isolated strains the Enterobacteriaceae (81.1%) *Escherichia coli* accounted for 45.4% and *Klebsiella pneumoniae* for 20.1%, followed by *Enterobacter cloacae* (5.2%), *Proteus mirabilis* (2.1%), *Citrobacter freundii* (1.8%), *Enterobacter aerogenes* (1.8%), *Klebsiella oxytoca* (1.4%), *Morganella morganii* (1.2%), *Serratia marcescens* (0.7%), *Citrobacter koseri* (0.3%), *Proteus vulgaris* (0.3%) and others (1.0%). Non- Enterobacteriaceae (18.9%) included *Pseudomonas aeruginosa* (9.8%), *Acinetobacter baumannii* (6.7%), *Stenotrophomonas maltophilia* (0.9%), *Aeromonas hydrophila* (0.4%) and others (1.1%). ESBL-screen positive *Escherichia coli* isolates (ESBL+) showed a decreasing trend from 67.5% in 2012 to 58.9% in 2014 of all *Escherichia coli* isolates and the percentage of ESBL+ *Klebsiella pneumoniae* isolates also decreased from 2012 through 2014 (40.4% to 26.6%), which was due to reduced percentages of ESBL+ isolates in HA IAIs for both bacteria. The overall susceptibilities of all 5160 IAI isolates were 87.53% to amikacin (AMK), 78.12% to piperacillin-tazobactam (TZP) 81.41% to imipenem (IMP) and 73.12% to ertapenem (ETP). The susceptibility of ESBL-screen positive *Escherichia coli* strains was 96.77%–98.8% to IPM, 91.26%–93.16% to ETP, 89.48%–92.75% to AMK and 84.86%–89.34% to TZP, while ESBL-screen positive *Klebsiella pneumoniae* strains were 70.56%–80.15% susceptible to ETP, 80.0%–87.5% to IPM, 83.82%–87.06% to AMK and 63.53%–68.38% to TZP within the three year study. Susceptibilities to all cephalosporins and fluoroquinolones were less than 50% beside 66.5% and 56.07% to cefoxitin (FOX) for ESBL+ *Escherichia coli* and *Klebsiella pneumoniae* strains respectively.

**Conclusions:**

The total ESBL+ rates decreased in *Escherichia coli* and *Klebsiella pneumoniae* IAI isolates due to fewer prevalence in HA infections. IPM, ETP and AMK were the most effective antimicrobials against ESBL+ *Escherichia coli* and *Klebsiella pneumoniae* IAI isolates in 2012–2014 and a change of fluoroquinolone regimens for Chinese IAIs is recommended.

**Electronic supplementary material:**

The online version of this article (10.1186/s12879-017-2873-z) contains supplementary material, which is available to authorized users.

## Background

The Study for Monitoring Antimicrobial Resistance Trends (SMART) is a global surveillance program, which monitors annually in vitro antimicrobial susceptibilities of hospital acquired (HA) and community acquired (CA) intra-abdominal and urinary tract infections due to aerobic and facultative Gram-negative bacilli (GNB). Intra-abdominal infections (IAIs) are the second most common cause of sepsis in intensive care units (ICU) [1] where they are the second most common cause of infection-related mortality [2]. IAIs are also the second most common cause of infection related to surgical interventions and according to a multicenter observational study in 68 medical institutions worldwide, [3] the overall mortality rate of patients with complicated IAIs in 2012–2013 was 10.5%, [4], with ESBL producing bacteria being a particular challenge for treatment [5]. However, initiation of appropriate antimicrobial therapy can significantly reduce the mortality rate of IAI-induced septic shock [6]. Since appropriate antibiotic therapy is essential for IAIs [7, 8], institutional and nationwide surveillance of IAI-derived bacterial strain susceptibilities provides crucial information for the selection of the right choice of empirical antimicrobial treatment.

Although a significant increase of the proportion of ESBL-positive *Enterobacteriaceae* hospital infections in Germany over the period 2007–2012 [9] and in Japan from 2000 to 2010 have been reported [10, 11] the situation in China is not clear. A limited number of ESBL-screen positive *Escherichia coli* and *Klebsiella pneumoniae* IAI isolates from 2012 and 2013 have been documented, but there is a wide diversity in ESBL-related molecular characteristics [12].

The present study mainly focused on ESBL-screen positive rates of IAI isolates and concomitantly on resistance rates of IAIs, in particularly those caused by *Enterobacteriaceae* against, 3rd and 4th generation cephalosporins, a cephamycin, 2nd generation fluoroquinolones, carbapenems, an aminoglycoside, as well as a combination of drugs containing penicillins with β-lactamase inhibitors. The data was collected from 21 centers in 16 Chinese cities between 2012 and 2014 in order to provide guidance for antimicrobial therapy of IAIs.

## Methods

### Collection and identification of isolates

The Human Research Ethics Committee of Peking Union Medical College Hospital approved this study and waived the need for consent (Ethics Approval Number: S-K238).

GNB strains were collected from consecutive IAI patients between 2012 and 2014 in 21 centers located in 16 Chinese cities. Only gram-negative aerobic and facultative anaerobic bacteria from abdominal infection sites such as the appendix, peritoneum, colon, bile, pelvis and pancreas were included and the strains needed to be pathogenic bacteria associated with clinical infections while gram-positive and anaerobic bacteria were excluded. The specimens were mainly obtained through surgical procedures, but puncture specimens such as intraperitoneal puncture fluid were also included and different gram-negative bacteria that were combined in one sample were also accepted. Exclusion criteria were isolates from drainage liquid or drainage bottles, as well as isolates from feces or perianal abscess environmental samples (not a patient source) or cultures for infection control purposes. All isolates were sent to the central clinical microbiology laboratory of Peking Union Medical College Hospital for initial bacteria identification and re-identification using MALDI-TOF MS (Vitek MS, BioMérieux, France). All organisms were considered clinically significant by local hospital criteria. Isolates collected within 48 h of hospitalization were categorized as CA IAIs, and those collected after 48 h were categorized as HA IAIs [13].

### Antimicrobial susceptibility test method

All isolate susceptibility tests and identification confirmations were carried out by the Clinical and Laboratory Standards Institute (CLSI) recommended broth microdilution method. Minimum inhibitory concentrations (MICs) interpretive criteria followed the 2014 M100-S24 guidelines of the CLSI. M100-S23 criteria were used to maintain the intermediate category for analysis [14]. Susceptibility to antimicrobial agents interpretations were based on clinical CLSI breakpoints, while the reference strains *Escherichia coli* ATCC 25922, *Pseudomonas aeruginosa* ATCC 27853 and *Klebsiella pneumoniae* ATCC 700603 were used as quality controls. Twelve antimicrobial agents were used for susceptibility tests in the present study, namely ceftriaxone (CRO), ceftazidime (CAZ), cefotaxime (CTX), cefepime (FEP), cefoxitin (FOX), ertapenem (ETP), imipenem (IPM), ampicillin-sulbactam (SAM), piperacillin-tazobactam (TZP), ciprofloxacin (CIP), levofloxacin (LVX) and amikacin (AMK).

### Extended-spectrum β-lactamase (ESBL) detection

Phenotypic identification of ESBL-screen positivity in *Escherichia coli* and *Klebsiella pneumoniae* (ESBL+) were carried out by CLSI recommended methods [15]. If cefotaxime or ceftazidime MICs were ≥2 μg/mL, the MICs of cefotaxime + clavulanic acid (4 μg/mL) or ceftazidime + clavulanic acid (4 μg/mL) were comparatively determined and ESBL production was defined as a ≥ 8-fold decrease in MICs for cefotaxime or ceftazidime tested in combination with clavulanic acid, compared to their MICs without clavulanic acid.

### Statistical analysis

All of the statistical analyses were performed using the IBM SPSS Statistics for Windows (Version 19.0. Armonk, NY: IBM Corp). The susceptibility of all Gram-negative isolates combined was calculated using breakpoints appropriate for each species and assuming 0% susceptible for species with no breakpoints for any given drug. The 95% confidence intervals were calculated using the adjusted Wald method; linear trends in susceptibility and ESBL rates were assessed for statistical significance using the Cochran-Armitage test; *P* values <0.05 were considered to be statistically significant.

## Results

### Basic information on IAI isolates collected from 2012 to 2014

From the included 5160 GNB strains (1917 strains were collected in 2012, 1665 strains in 2013, and 1578 strains in 2014), the majority (79.8–83.8%) belonged to *Enterobacteriaceae* including *Escherichia coli* (45.4% of all GNBs), *Klebsiella pneumoniae* (20.1% of all GNBs), followed by *Enterobacter cloacae* (5.2%), *Proteus mirabilis* (2.1%), *Citrobacter freundii* (1.8%), *Enterobacter aerogenes* (1.8%), *Klebsiella oxytoca* (1.4%), *Morganella morganii* (1.2%), *Serratia marcescens* (0.7%), *Citrobacter koseri* (0.3%), *Proteus vulgaris* (0.3%) and others 1.0%. Non-*Enterobacteriaceae* were isolated from 16.2–20.2% of all GNB caused IAIs and included *Pseudomonas aeruginosa* (9.8%), *Acinetobacter baumannii* (6.7%), *Stenotrophomonas maltophilia* (0.9%), *Aeromonas hydrophila* (0.4%) and others (1.1%) (Additional file 1: Table S1). Overall susceptibilities of the 5160 IAIs to the 12 antimicrobial agents tested in the study are shown in Table 1. Highest overall susceptibilities of the 5610 GNB isolates from IAIs between 2012 and 2014 were found to amikacin (87.53%), imipenem (81.41%), piperacillin tazobactam (78.12%) and ertapenem (73.12%). Susceptibilities to all tested cephalosporins, fluoroquinolones and ampicillin sulbactam were between 22.24% and 58.75%.

**Table 1:** Susceptibilities of all 5160 included strains derived from Chinese IAIs from 2012 to 2014 to 12 tested antibiotics

	Percent Susceptibilities			
	2012	2013	2014	Sum
Amikacin	87.20	89.16	86.22	87.53
Piperacillin Tazobactam	76.77	81.85	75.75	78.12
Ampicillin Sulbactam	18.70	24.72	23.30	22.24
Imipenem	80.66	84.26	79.30	81.41
Ertapenem	73.18	75.71	70.48	73.12
Cefoxitin	50.00	55.52	52.38	52.64
Ceftazidime	54.80	61.77	59.69	58.75
Cefepime	43.67	51.92	51.05	48.88
Ceftriaxone	31.25	36.74	38.66	35.55
Cefotaxime	30.98	36.49	37.46	34.98
Levofloxacin	49.63	53.90	55.62	53.05
Ciprofloxacin	45.52	48.20	50.35	48.02

The relative percentages of ESBL-screen positive *Escherichia coli* and *Klebsiella pneumoniae* strains from IAI isolates showed a decreasing trend from 2012 to 2014 for *Klebsiella pneumoniae* (*P* = 0.021) and for *Escherichia coli* (67.5% to 58.9%), though not significant for the later one (Table 2).

**Table 2:** ESBL-screen positive strain percentages of HA and CA derived from IAIs (n/n, %)

Organism			2012 *N* (%)	2013 *N* (%)	2014 *N* (%)	Total *N* (%)	*P-*value
ESBL+ strains in IAIs		Total	767/1917 (40.0)	626/1665 (37.6)^a^	507/1578 (32.1)^a^	1900/5160 (36.8)^a^	0.0038
		CA	166/465 (35.7)	121/375 (32.3)	204/598 (34.1)	491/1438 (34.1)	0.7651
		HA	601/1452 (41.4)	503/1277 (39.4)	295/959 (30.8)	1399/3688 (37.9)	0.0011
*E. coli* ESBL+	Of all *E. coli*	Total	599/887 (67.5)	469/772 (60.8)^a^	403/684 (58.9)^a^	1471/2343 (62.8)^a^	0.1978
		CA	138/234 (59.0)	99/192 (51.6)	162/269 (60.2)	399/695 (57.4)	0.5920
		HA	461/653 (70.6)	368/575(64.0)	236/406 (58.1)	1065/1634 (65.2)	0.1528
	Of all ESBL+ IAI isolates		599/767 (78.1)	469/626 (74.9)	403/507 (79.5)	1471/1900 (77.4)	0.7905
*K. pneumoniae* ESBL+	Of all *K. pneumoniae*	Total	136/337 (40.4)	145/381 (38.1)	85/319 (26.6)^a^	366/1037 (35.3)^a^	0.0215
		CA	27/87 (31.0)	21/85 (24.7)	33/112 (29.5)	81/284 (28.5)	0.7703
		HA	109/250 (43.6)	124/295 (42.0)	49/201 (24.4)	282/746 (37.8)	0.0060
	Of all ESBL+ IAI isolates		136/767 (17.7)	145/626 (23.2)	85/507 (16.8)	366/1900 (19.3)	0.0448
*P. mirabilis* ESBL+	Of all *P. mirabilis*	Total	24/48 (50.0)	8/32 (25.0)	12/29 (41.4)	44/109 (40.4)	0.3265
		CA	0/11 (0.0)	1/10 (10.0)	3/9 (33.3)	4/30 (13.3)	0.1680
		HA	24/37 (64.9)	7/22 (31.8)	9/20 (45.0)	40/79 (50.6)	0.3411
	Of all ESBL+ IAI isolates		24/767 (3.1)	8/626 (1.3)	12/507 (2.4)	44/1900 (2.3)	0.1715
*K. oxytoca* ESBL+	Of all *K. oxytoca*	Total	8/21 (38.1)	4/18 (22.2)	7/31 (22.6)	19/70 (27.1)^a^	0.6073
		CA	1/3 (33.3)	0/5 (0)	6/19 (31.6)	7/27 (25.9)	0.4672
		HA	7/18 (38.9)	4/12 (33.3)	1/12 (8.3)	12/42 (28.6)	0.3426
	Of all ESBL+ IAI isolates		8/767 (1.0)	4/626 (0.6)	7/507 (1.4)	19/1900 (1.0)	0.4608

Note: *Escherichia coli* (*E. coli*), *Klebsiella pneumoniae* (*K. pneumoniae*), Hospital Acquired (HA), Community Acquired (CA)

^a^indicates that few strains were not categorized into HA and CA IAIs

Of all ESBL-screen positive bacterial strains isolated from IAIs (1900), *Escherichia coli* ESBL+ strains were the most frequently isolated (74.9–79.5% of all ESBL+ IAIs), followed by *Klebsiella pneumoniae* (16.8–23.2% of all ESBL+ IAIs) and *Proteus mirabilis* (1.3–3.1% of all ESBL+ IAIs) with the least frequent isolated ESBL+ strain being *Klebsiella oxytoca* with only 0.6–1.4% of all ESBL producing strains isolated from IAIs between 2012 and 2014. The overall percentages of ESBL production in the *Escherichia coli*, *Klebsiella pneumoniae*, *Proteus mirabilis* and *Klebsiella oxytoca* IAI strains was 36.8% with fairly constant rates for *Escherichia coli*, *Proteus mirabilis* and *Klebsiella oxytoca,* but a significant decrease of ESBL producing HA *Klebsiella pneumoniae* strains during this period (from 43.6% and 42.0% in 2012 and 2013 to 24.4% in 2014), which also reflected in a significant overall ESBL+ reduction in *Escherichia coli*, *Klebsiella pneumoniae*, *Proteus mirabilis* and *Klebsiella oxytoca* IAI isolates from 40.0% in 2012 to 37.6% in 2013 and to 32.1% in 2014 (*P* = 0.0038) and the overall ESBL+ production changes of *Klebsiella pneumoniae* within the 3 year observation period (*P* = 0.0215) (Table 2).

We next investigated the source of GNB strains isolated from IAIs (gall bladder, peritoneal fluid, abscess, appendix, liver and pancreas). The most frequently infected organ was the gall bladder (1072), followed by peritoneal fluid (812) and abscesses (650). The least infected organ was the pancreas (85). *Escherichia coli* infections occurred more frequently than *Klebsiella pneumoniae* infections in all organs beside the liver, in which the number of *Klebsiella pneumoniae* isolates (148) was higher than the *Escherichia coli* (88) isolates. In addition, over the three years, the highest percentages of ESBL-screen positive *Escherichia coli* and *Klebsiella pneumoniae* occurred in pancreatic isolates*.* In accordance with the general ESBL+ percentage decrease in *Escherichia coli*, isolates from the main 6 organs collected in 2014 contained less ESBL + −producing *Escherichia coli* strains than in 2012, which occurred for *Klebsiella pneumoniae* only in gall bladder, abscess, liver and appendix infections (Additional file 2: Figure S1).

### In vitro susceptibility of ESBL-screen positive *Escherichia coli* and *Klebsiella pneumoniae* strains isolated from IAIs during 2012–2014

**Fig. 1:**
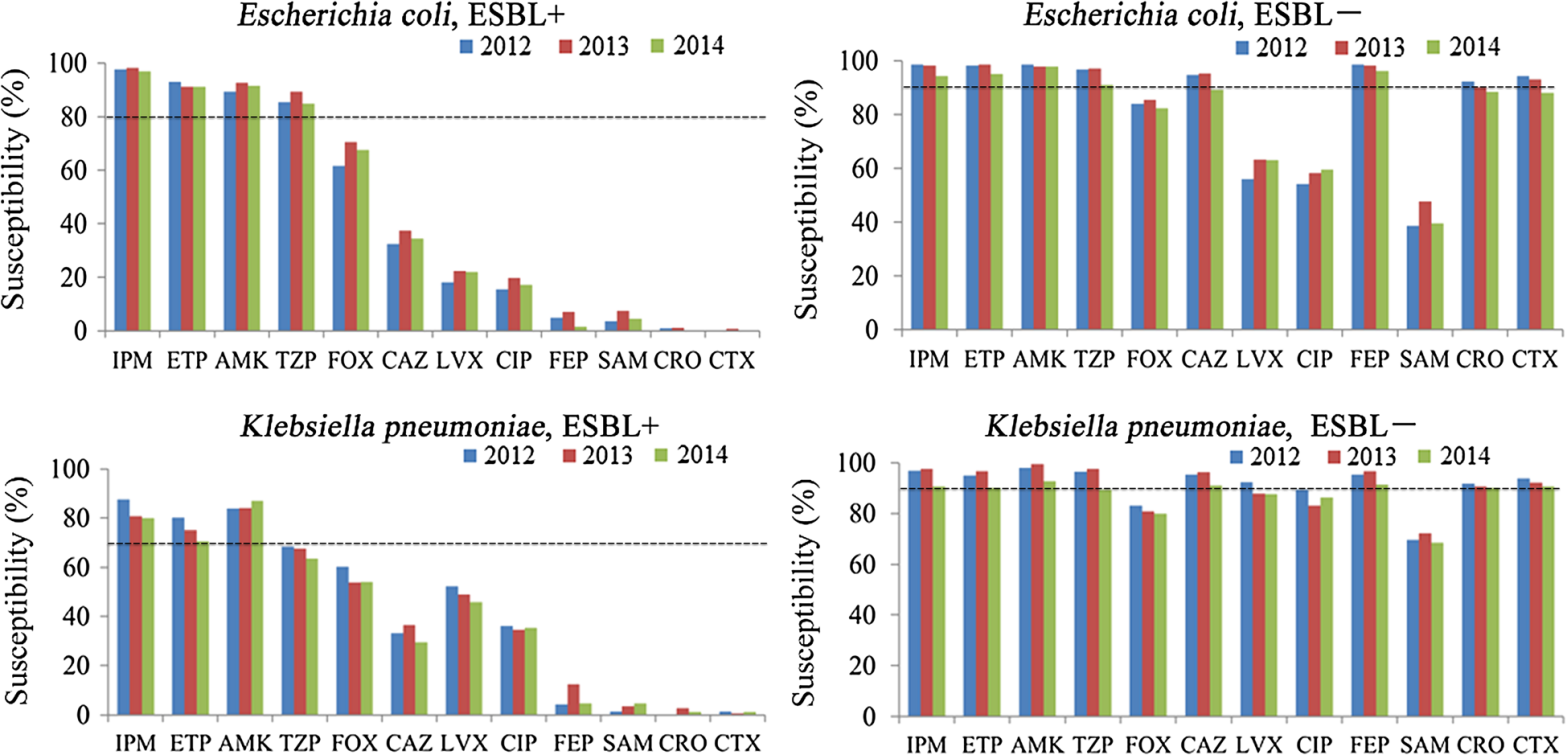
In vitro antimicrobial susceptibilities of ESBL+ or non-ESBL-screen positive *Escherichia coli* strains and ESBL+ or non-ESBL-screen positive *Klebsiella pneumoniae* strains causing IAIs between 2012 and 2014

Susceptibilities of ESBL-screen positive *Escherichia coli* strains were 96.66%- 98.08% to IPM, 91.26%–93.16% to ETP, 89.48%–92.75% to AMK and 84.86%- 89.34% to TZP, whereas susceptibilities to FOX was 61.60%–70.58% and varied for CAZ, LVX, CIP, FEP, SAM, CRO and CTX between 0% and 37.53%. ESBL-screen positive *Klebsiella pneumoniae* strains were 70.59%–80.15% susceptible to ETP, 80.0%–87.5% to IPM and 83.82%–87.06% to AMK as well as 53.79%–60.29% to FOX and also varied between 0% and 52.21% for CAZ, LVX, CIP, FEP, SAM,CRO and CTX within the three years of our study. The susceptibility of non-ESBL-screen positive *Escherichia coli* and non-ESBL-screen positive *Klebsiella pneumoniae* strains were (94.27%–98.55%) and (90.52%–97.65%) to IPM, (94.98%–98.55% and 90.09%–96.71%) to ETP, (97.82%-98.55%) and (92.67%-99.53%) to AMK, (91.04%–97.09%) and (89.22%–97.65%) to TZP, (82.44%–85.4%) and (79.74%–82.99%) to FOX, (56.0%- 63.27%) and (87.5%–92.27%) to LVX, (89.25%–95.27%) and 90.95%–96.24%) to CAZ, (96.06%–98.55%) and (91.38%–96.71%) to FEP, (88.53%–92.36%) and (90.09%–91.75%) to CRO, as well as (88.17%–94.18%) and (90.52%–93.18%) to CTX, whereas susceptibilities to SAM were only (39.43%–47.64%) and (68.53%–72.3%), respectively (Fig. 1).

**Fig. 2:**
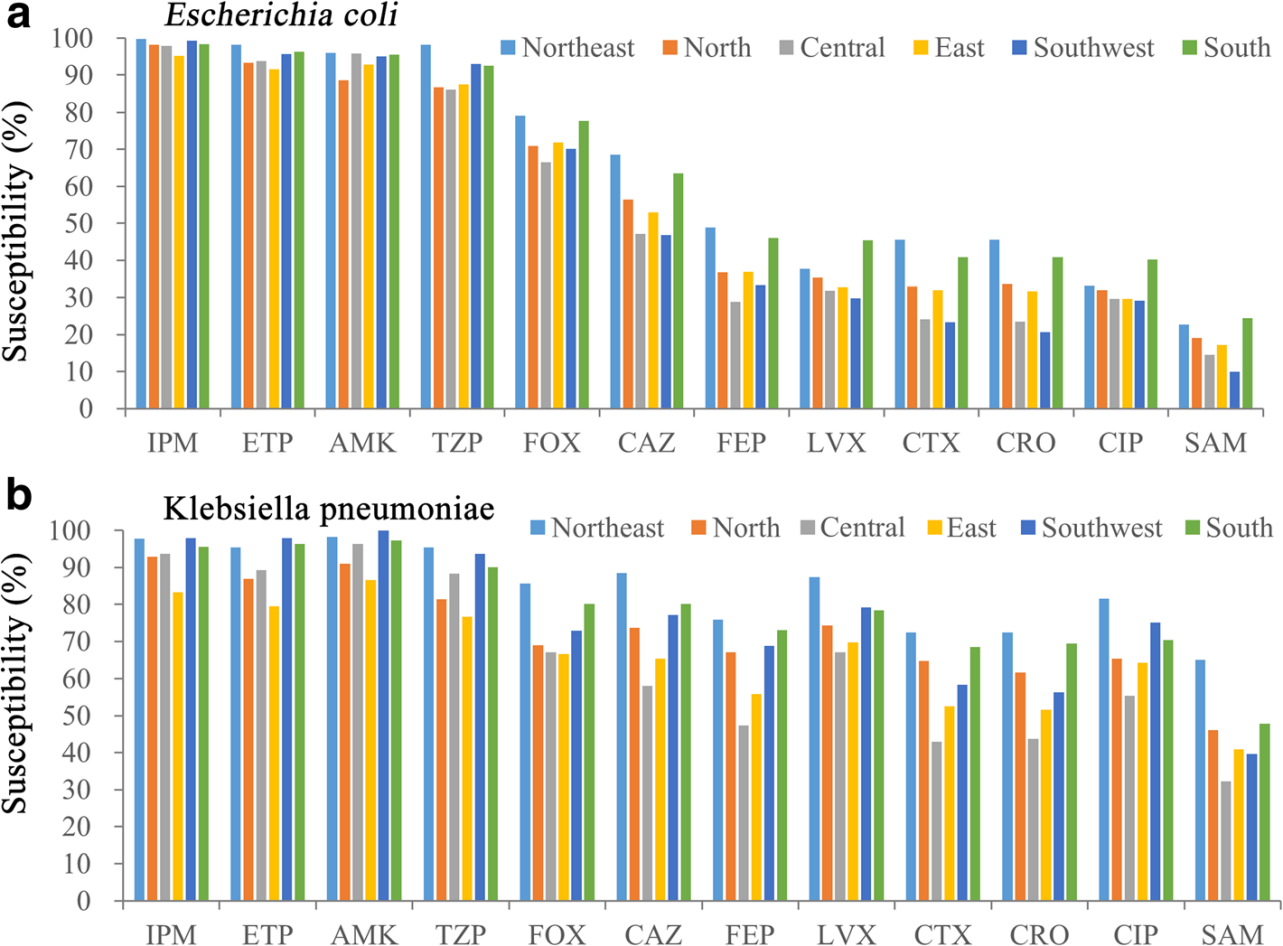
Antimicrobial susceptibilities for *Escherichia coli* and *Klebsiella pneumoniae* IAI isolates from the northeastern, northern, central, eastern, southwestern and southern regions of China to the indicated antimicrobials

Next, we investigated local differences of susceptibilities to 12 antibiotics **(**Fig. 2) and the susceptibility rates against *Escherichia coli* and *Klebsiella pneumoniae* were essentially higher in participating centers located in the south and northeast compared to the southwest and central China. In addition, the susceptibility rates to IPM ETP AMK and TZP of *Klebsiella pneumoniae* were lowest in the centers from the east of China, indicating the development of multi-resistant strains in this region.

There was no significant difference in antimicrobial susceptibility among the *Escherichia coli* and *Klebsiella pneumoniae* IAIs producing ESBL strains between HA and CA infections. However there was a tendency that susceptibility of *Klebsiella pneumoniae* ESBL+ infections was lower in CA than in HA IAIs (Table 3).

**Table 3:** The susceptibilities of ESBL-screen positive *Escherichia coli* and *Klebsiella pneumoniae* strains isolated from HA and CA IAIs

	*Escherichia coli*, ESBL+		*Klebsiella pneumoniae*, ESBL+	
	HA%	CA%	HA%	CA%
Amikacin	91.46	89.47	86.17	80.25
Piperacillin Tazobactam	85.73	88.72	67.37	66.67
Ampicillin Sulbactam	4.70	5.76	3.19	2.47
Imipenem	97.84	96.74	84.40	80.25
Ertapenem	92.02	91.98	77.66	71.61
Cefoxitin	65.63	67.42	59.58	45.68
Ceftazidime	33.80	37.09	36.17	25.93
Cefepime	4.60	4.76	8.51	4.94
Ceftriaxone	0.75	0.75	1.77	0.00
Cefotaxime	0.47	0.00	1.06	1.23
Levofloxacin	19.91	22.56	50.00	49.38
Ciprofloxacin	17.09	18.04	36.17	33.33

## Discussion

The majority of IAI isolates collected in the participating centers consisted of *Escherichia coli* and *Klebsiella pneumoniae* which is similar with data from the 2002 to 2009 SMART study [16]. However, in contrast to the 2002–2009 SMART study data, which revealed an increase particularly of ESBL-screen positive *Escherichia coli* strains from 20.8% in 2002 up to 64.9% in 2009, in the present study ESBL+ rates in *Escherichia coli* strains decreased from 67.5% in 2012 to 58.9% in 2014, which was reflected also in gall bladder, abscess, liver, peritoneal fluid, appendix and pancreas derived isolates sampled in 2014. In the present study the percentages of ESBL+ *Klebsiella pneumoniae* strains notably dropped from 40.4% in 2012 to 26.6% in 2014 (*P* = 0.0215) (Table 2), which was also seen in the decreased ESBL+ percentages of isolates from the gall bladder, abscess, liver and appendix. However, the percentages of ESBL+ *Escherichia coli* (66.7%) and *Klebsiella pneumoniae* (55.6%) strains isolated from pancreas remained high in 2014. Most of the infections occurred in the gall bladder and the peritoneum, which is in accordance with previous literature, but the number of isolates from the appendix was unusually low in our study [4, 17]. In contrast to other organs, the number of *Klebsiella pneumoniae* liver infections exceeded those caused by *Escherichia coli*, which has also been reported in previous studies, and might be explained by cryptogenic infections with a new hypervirulent K1 *Klebsiella pneumoniae* ST23 strain, which developed in Asia and spread to Australia, European countries and the USA [18–20], while a recent report by Qu et al. (2015) et al. noted that K1 ST23 were the predominant *Klebsiella pneumoniae* liver abscess causing strains in east China [21]. ESBL-producing *Escherichia coli* and *Klebsiella pneumoniae* are supposed to be susceptible to cefoxitin, but a high proportion of these isolates tested cefoxitin-resistant (Table 3) and a likely explanation is that they have acquired AmpC beta-lactamases and porin loss, which has been described in a previous study about cefoxitin resistant *Klebsiella pneumoniae* strains in China, which expressed DHA-1 ß-lactamase combined with porin OmpK36 deficiency [22].

The percentages of *Escherichia coli* and *Klebsiella pneumoniae* ESBL+ strains was higher in HA than in CA IAI isolates, which is in accordance with a previous Chinese SMART study by Yang et al. (2013). However, in the latter study *Escherichia coli* ESBL+ rates in CA infections constantly rose from 19.1% in 2002–2003 to 61.6% in 2010–2011, whereas in our study the *Escherichia coli* ESBL+ rates in CA IAIs were relative constant at around 60%, with a reduction to 51.6% only in 2013. In contrast, the *Escherichia coli* ESBL+ rates in HA IAIs showed a decreasing trend from 70.6% in 2012 to 58.1% in 2014 in our study, but were relative stable in the years 2006–2011 (66.7%–70.0%) [23].

A more dramatic change was visible for *Klebsiella pneumoniae* ESBL+ rates in HA IAIs dropping from 43.6% in 2012 and 42.0% in 2013, which is similar to the Chinese HA values (39.4%) reported for *Klebsiella pneumoniae* IAIs in 2010–2011 [23], to 24.4% in 2014 *(P* = 0.006), but in CA IAIs the *Klebsiella pneumoniae* ESBL+ rates were relatively constant (between 24.7% and 31.0%), which is somewhat higher than the 22.2% reported for CA *Klebsiella pneumoniae* IAIs in 2010–2011 [23]. Taken together, the total ESBL+ rates in *Klebsiella pneumoniae* and *Escherichia coli* isolates from IAIs in our study dropped between 2012 and 2014, which was due to less ESBL+ rates in HA IAIs and rather constant percentages of CA ESBL+ *Klebsiella pneumoniae* and *Escherichia coli* IAI isolates (Table 2). The decrease of ESBL+ GNBs might be explained by new restrictions for the clinical application of antimicrobial agents, which has been introduced by the Chinese ministry of health in 2012 [24]. The overall susceptibilities of ESBL positive *Escherichia coli* strains was 96.77%–98.8% to IPM, 91.26%–93.16% to ETP, 89.48%–92.75% to AMK and 84.86%–89.34% to TZP, while ESBL-screen positive *Klebsiella pneumoniae* strains were 70.56% -80.15% susceptible to ETP, 80.0%–87.5% to IPM, 83.82%–87.06% to AMK and 63.53%–68.38% to TZP within the three year study. However, it is noteworthy that reduced susceptibilities of *Klebsiella pneumonia* strains to the carbapenems IPM and ETP derived from centers located in east China indicated a local carbapenem-resistance, which has also been described in other countries [25, 26]. Because the eastern part of China is the most developed region with the highest incomes, antimicrobial overuse [27, 28] might be an explanation for the carbapenem susceptibility difference in this area, which has been described also for the eastern Zhejiang Province before [29]. In general a previous study noted that Chinese individuals were harboring the highest number and abundance of antibiotic resistance genes in their gut microbiota compared to Danish and Spanish individuals [30], but we did not include investigations of molecular mechanisms of resistances.

All tested cephalosporins and fluoroquinolones were <70% effective for *Escherichia coli* and <60% for *Klebsiella pneumoniae* isolates that produced ESBLs. This finding is in agreement with previous literature that suggested that carbapenems are the best choice as empirical mono therapies, especially for complicated IAIs [31], but that cephalosporins, fluoroquinolones and SAM are not ideal choices for empirical treatment of IAIs in China [16]. For non-ESBL-screen positive *Escherichia coli* strains, cefoxitin, levofloxacin, ciprofloxacin and ampicillin-sulbactam, and for non-ESBL-screen positive *Klebsiella pneumoniae* strains, cefoxitin and ampicillin-sulbactam were the least effective antibiotics (Fig. 1).

Taken together, the relatively high susceptibility percentages seen for imipenem and, to a slightly lesser degree, ertapenem, against *Escherichia coli* —whether ESBL-positive or -negative—are important considerations in China, where ESBL+ rates around 60% are seen, and many other drugs from the beta-lactam class and fluoroquinolones are no longer viable options for therapy and should be avoided unless susceptibilities to this antimicrobial agents have been confirmed. Against *Klebsiella pneumoniae*, the carbapenem activity is somewhat lower, presumably because of presence of more carbapenemases (such as KPC-2-type) in *Klebsiella pneumoniae* [32, 33], as well as other mechanisms that include porin loss combined with AmpC or ESBL enzymes; however, even the reduced activity of carbapenems to *Klebsiella pneumoniae* is dramatically higher than all other drugs evaluated in SMART except for amikacin and piperacillin-tazobactam. Considering the relatively high ESBL rates in China, and the low susceptibility to fluoroquinolones that are usually seen in conjunction with ESBL-positive isolates, carbapenems are among the few antimicrobial agents in China retaining sufficient in vitro activity to be considered for empiric therapy. On the other hand, it is very important to retain the activity of carbapenems, so step-down therapy to other agents should always be considered once the susceptibility of a specific pathogen is known.

A limitation of the study was that genotypic or molecular data of the strains were not included, since the SMART project does not involve these kind of analyzes.

## Conclusions

From 2012 to 2014, a total of 5160 IAI isolates were obtained, of which 81.1% were caused by *Enterobacteriaceae* and 18.9% by non-*Enterobacteriaceae*, with *Escherichia coli* (45.4%) being the most common followed by *Klebsiella pneumoniae* (20.1%). The most common non-*Enterobacteriaceae* were *Pseudomonas aeruginosa* (9.8%) and *Acinetobacter baumannii* (6.7%). The percentages of ESBL-screen positive *Escherichia coli* and *Klebsiella pneumoniae* strains in IAI GNB isolates showed a decreasing trend from 2012 to 2014, which can be explained by less ESBL+ percentages in strains from HA IAIs.

Susceptibility of ESBL-screen positive *Escherichia coli* strains was >80% to imipenem, ertapenem, amikacin and piperacillin-tazobactam, while ESBL-screen positive *Klebsiella pneumoniae* strains were >70% susceptible only to imipenem, ertapenem and amikacin.

In contrast to gall bladder, abscess, peritoneal fluid, appendix and pancreas, the percentage of *Klebsiella pneumoniae* causing liver infections was higher than that caused by *Escherichia coli*. It is noteworthy that *Klebsiella pneumoniae* and *Escherichia coli* isolates from pancreatic infections exhibited consistently high ESBL+ rates.

The apparent trend of declining percentages of ESBL-screen positive *Escherichia coli* and *Klebsiella pneumoniae* strains needs to be closely monitored.

## Additional files

Additional file 1: Table S1. Bacterial identification and epidemiological status of isolates from intra-abdominal infections in China (2012–2014). (DOC 54 kb)

Additional file 2: Figure S1. Sources of ESBL-screen positive *Escherichia coli* and *Klebsiella pneumoniae* IAI isolates from 2012 to 2014. The upper numbers indicate the total number of isolates and the grey areas and numbers in the grey areas of the bars indicate the percentage of ESBL+ strains. (TIFF 748 kb)

## Abbreviations

AMK: Amikacin
CA: Community acquired
CAZ: Ceftazidime
CIP: Ciprofloxacin
CLSI: Clinical and Laboratory Standards Institute
CRO: Ceftriaxone
CTX: Cefotaxime
EPM: Ertapenem
ESBL: Extended spectrum beta-lactamase
FEP: Cefepime
FOX: Cefoxitin
GNB: Gram-negative bacterial
HA: Hospital acquired
IAIs: Intra-abdominal infections
ICU: Intensive care units
IPM: Imipenem
LVX: Levofloxacin
MICs: Minimum inhibitory concentrations
SAM: Ampicillin-sulbactam
SMART: Study for Monitoring Antimicrobial Resistance Trends
TZP: Piperacillin-tazobactam

## References

[1] Lopez N, Kobayashi L, Coimbra RA (2011). Comprehensive review of abdominal infections. World J Emerg Surg..

[2] Hoffmann C, Zak M, Avery L, Brown J. Treatment modalities and antimicrobial stewardship initiatives in the Management of Intra-Abdominal Infections. Antibiotics (Basel). 2016;5 10.3390/antibiotics5010011 PMC4810413 27025526

[3] Sharma D, Hayman K, Stewart BT, Dominguez L, Trelles M, Saqeb S (2015). Surgery for conditions of infectious etiology in resource-limited countries affected by crisis: the Medecins sans Frontieres operations Centre Brussels experience. Surg Infect.

[4] Sartelli M, Catena F, Ansaloni L, Coccolini F, Corbella D, Moore EE (2014). Complicated intra-abdominal infections worldwide: the definitive data of the CIAOW study. World J Emerg Surg..

[5] Boontham P, Soontomrak R (2015). Intra-abdominal infections: prevalence and risk factors of ESBLs infections. J Med Assoc Thail.

[6] Kumar A, Ellis P, Arabi Y, Roberts D, Light B, Parrillo JE (2009). Initiation of inappropriate antimicrobial therapy results in a fivefold reduction of survival in human septic shock. Chest.

[7] Chong YP, Bae IG, Lee SR, Chung JW, Jun JB, Choo EJ (2015). Clinical and economic consequences of failure of initial antibiotic therapy for patients with community-onset complicated intra-abdominal infections. PLoS One.

[8] Edelsberg J, Berger A, Schell S, Mallick R, Kuznik A, Oster G (2008). Economic consequences of failure of initial antibiotic therapy in hospitalized adults with complicated intra-abdominal infections. Surg Infect.

[9] Leistner R, Schroder C, Geffers C, Breier AC, Gastmeier P, Behnke M (2015). Regional distribution of nosocomial infections due to ESBL-positive Enterobacteriaceae in Germany: data from the German National Reference Center for the Surveillance of Nosocomial Infections (KISS). Clin Microbiol Infect.

[10] Hara T, Sato T, Horiyama T, Kanazawa S, Yamaguchi T, Maki H (2015). Prevalence and molecular characterization of CTX-M extended-spectrum beta-lactamase-producing Escherichia Coli from 2000 to 2010 in Japan. Jpn J Antibiot.

[11] Sato T, Hara T, Horiyama T, Kanazawa S, Yamaguchi T, Maki H (2015). Mechanism of resistance and antibacterial susceptibility in extended-spectrum beta-lactamase phenotype Klebsiella Pneumoniae and Klebsiella oxytoca isolated between 2000 and 2010 in Japan. J Med Microbiol.

[12] Liao K, Chen Y, Wang M, Guo P, Yang Q, Ni Y (2017). Molecular characteristics of extended-spectrum beta-lactamase-producing Escherichia Coli and Klebsiella Pneumoniae causing intra-abdominal infections from 9 tertiary hospitals in China. Diagn Microbiol Infect Dis.

[13] Hawser SP, Bouchillon SK, Hoban DJ, Badal RE (2009). Vitro susceptibilities of aerobic and facultative anaerobic gram-negative bacilli from patients with intra-abdominal infections worldwide from 2005-2007: results from the SMART study. Int J Antimicrob Agents.

[14] CLSI. Performance Standards for Antimicrobial Susceptibility Testing; Twenty-Fourth Informational Supplement. CLSI document M100-S24. Wayne, PA: Clinical and Laboratory Standards Institute;. 2014.

[15] CLSI. Performance Standards for Antimicrobial Susceptibility. Testing; 22nd Informational Supplement M100-S22. Wayne, PA: Clinical and Laboratory Standards Institute. 2012.

[16] Yang Q, Wang H, Chen M, Ni Y, Yu Y, Hu B (2010). Surveillance of antimicrobial susceptibility of aerobic and facultative gram-negative bacilli isolated from patients with intra-abdominal infections in China: the 2002-2009 study for monitoring antimicrobial resistance trends (SMART). Int J Antimicrob Agents.

[17] Sartelli M, Catena F, Ansaloni L, Moore E, Malangoni M, Velmahos G (2013). Complicated intra-abdominal infections in a worldwide context: an observational prospective study (CIAOW study). World J Emerg Surg.

[18] Liu Y, Wang JY, Jiang W (2013). An increasing prominent disease of Klebsiella Pneumoniae liver abscess: etiology, diagnosis, and treatment. Gastroenterol Res Pract.

[19] Siu LK, Fung CP, Chang FY, Lee N, Yeh KM, Koh TH (2011). Molecular typing and virulence analysis of serotype K1 Klebsiella Pneumoniae strains isolated from liver abscess patients and stool samples from noninfectious subjects in Hong Kong, Singapore, and Taiwan. J Clin Microbiol.

[20] Lubbert C, Wiegand J, Karlas T (2014). Therapy of liver abscesses. Viszeralmedizin.

[21] TT Q, Zhou JC, Jiang Y, Shi KR, Li B, Shen P (2015). Clinical and microbiological characteristics of Klebsiella Pneumoniae liver abscess in East China. BMC Infect Dis.

[22] Shi W, Li K, Ji Y, Jiang Q, Wang Y, Shi M (2013). Carbapenem and cefoxitin resistance of Klebsiella Pneumoniae strains associated with porin OmpK36 loss and DHA-1 beta-lactamase production. Braz J Microbiol.

[23] Yang Q, Zhang H, Wang Y, Xu Y, Chen M, Badal RE (2013). A 10 year surveillance for antimicrobial susceptibility of Escherichia Coli and Klebsiella Pneumoniae in community- and hospital-associated intra-abdominal infections in China. J Med Microbiol.

[24] China. MoHotPsRo. Management of the clinical application of antimicrobial agents (Ministry of Health Order No. 84). 2012.

[25] Vatopoulos A. High rates of metallo-beta-lactamase-producing Klebsiella Pneumoniae in Greece--a review of the current evidence. Euro Surveill. 2008;13 18445397

[26] Bratu S, Landman D, Haag R, Recco R, Eramo A, Alam M (2005). Rapid spread of carbapenem-resistant Klebsiella Pneumoniae in new York City: a new threat to our antibiotic armamentarium. Arch Intern Med.

[27] Curcio D (2011). Off-label use of antibiotics in intensive care unit: the multidrug-resistant pathogens challenge. J Crit Care.

[28] Jensen US, Skjot-Rasmussen L, Olsen SS, Frimodt-Moller N, Hammerum AM, Group DS (2009). Consequences of increased antibacterial consumption and change in pattern of antibacterial use in Danish hospitals. J Antimicrob Chemother.

[29] Zhang R, Ichijo T, YY H, Zhou HW, Yamaguchi N, Nasu M, et al. A ten years (2000-2009) surveillance of resistant Enterobacteriaceae in Zhejiang Province, China. Microb Ecol Health Dis. 2012;23 10.3402/mehd.v23i0.11609 PMC3744663 23990814

[30] Hu Y, Yang X, Qin J, Lu N, Cheng G, Wu N (2013). Metagenome-wide analysis of antibiotic resistance genes in a large cohort of human gut microbiota. Nat Commun.

[31] Solomkin JS, Mazuski JE, Bradley JS, Rodvold KA, Goldstein EJ, Baron EJ (2010). Diagnosis and management of complicated intra-abdominal infection in adults and children: guidelines by the surgical infection society and the Infectious Diseases Society of America. Clin Infect Dis.

[32] Lou Z, Qi Y, Qian X, Yang W, Wei Z (2014). Emergence of Klebsiella Pneumoniae carbapenemase-producing Escherichia Coli sequence type 131 in Hangzhou, China. Chin Med J.

[33] Chen S, Hu F, Xu X, Liu Y, Wu W, Zhu D (2011). High prevalence of KPC-2-type carbapenemase coupled with CTX-M-type extended-spectrum beta-lactamases in carbapenem-resistant Klebsiella Pneumoniae in a teaching hospital in China. Antimicrob Agents Chemother.

