# Supplementary material for: Update of incidence and antimicrobial susceptibility trends of Escherichia coli and Klebsiella pneumoniae isolates from Chinese intra-abdominal infection patients
Source: BMC Infect Dis. 2017 Dec 18;17:776. doi: 10.1186/s12879-017-2873-z (PMC5735800; doi:10.1186/s12879-017-2873-z)
Supplement: Supplementary file 1 — Bacterial identification and epidemiological status of isolates from intra-abdominal infections in China (2012–2014). (DOC 54 kb) [file 12879_2017_2873_MOESM1_ESM.doc]

Supplemental Table 1. Bacterial identification and epidemiological status of isolates from intra-abdominal infections in China (2012-2014)

| **Organism (% of GNBs)** | **Sum N (%)** | **2012 N (%)** | **2013 N (%)** | **2014 N (%)** | ***P*-value** |
| --- | --- | --- | --- | --- | --- |
| **Total GNB** | **5,160 (100)** | **1,917 (100)** | **1,665 (100)** | **1,578 (100)** | **< 0.0001** |
| ***Enterobacteriaceae*** | **4,186 (81.1)** | **1,529 (79.8)** | **1,395 (83.8)** | **1,262 (80.0)** | **0.5551** |
| *Escherichia coli* | 2,343 (45.4) | 887 (46.3) | 772 (46.4) | 684 (43.4) | 0.4782 |
| ESBL-screen positive strains | 1,471/2,343 (62.8) | 599/887 (67.5) | 469/772 (60.8) | 403/684 (58.9) | 0.1978 |
| Non-ESBL strains | 829/2,343 (35.4) | 275/887 (31.0) | 275/772 (35.6) | 279/684 (40.8) | 0.0214 |
| Not identified | 43/2,343 (1.8) | 13/887 (1.5) | 28/772 (3.6) | 2/684 (0.3) | < 0.0001 |
| *Klebsiella pneumoniae* | 1,037 (20.1) | 337 (17.6) | 381 (22.9) | 319 (20.2) | 0.0056 |
| ESBL-screen positive strains | 366/1,037 (35.3) | 136/337 (40.4) | 145/381 (38.1) | 85/319 (26.6) | 0.0215 |
| Non-ESBL strains | 639/1,037 (61.6) | 194/337 (57.6) | 213/381 (55.9) | 232/319 (72.7) | 0.0619 |
| Not identified | 32/1,037 (3.1) | 7/337 (2.1) | 23/381 (6.0) | 2/319 (0.6) | 0.0002 |
| *Enterobacter cloacae* | 266 (5.2) | 101 (5.3) | 85 (5.1) | 80 (5.1) | 0.9634 |
| *Proteus mirabilis* | 109 (2.1) | 48 (2.5) | 32 (1.9) | 29 (1.8) | 0.3344 |
| *Citrobacter freundii* | 95 (1.8) | 33 (1.7) | 30 (1.8) | 32 (2.0) | 0.7972 |
| *Enterobacter aerogenes* | 91 (1.8) | 37 (1.9) | 23 (1.4) | 31 (2.0) | 0.3660 |
| *Klebsiella oxytoca* | 70 (1.4) | 21 (1.1) | 18 (1.1) | 31 (2.0) | 0.0476 |
| *Morganella morganii* | 63 (1.2) | 15 (0.8) | 24 (1.4) | 24 (1.5) | 0.0910 |
| *Serratia marcescens* | 34 (0.7) | 15 (0.8) | 9 (0.5) | 10 (0.6) | 0.6676 |
| *Citrobacter koseri* | 14 (0.3) | 3 (0.2) | 5 (0.3) | 6 (0.4) | 0.4341 |
| *Proteus vulgaris* | 13 (0.3) | 8 (0.4) | 3 (0.2) | 2 (0.1) | 0.1834 |
| Other | 51 (1.0) | 24 (1.3) | 13 (0.8) | 14 (0.9) | 0.3307 |
| ***Non-Enterobacteriaceae*** | **974 (18.9)** | **388 (20.2)** | **270 (16.2)** | **316 (20.0)** | **0.0196** |
| *Pseudomonas aeruginosa* | 505 (9.8) | 207 (10.8) | 141 (8.5) | 157 (10.0) | 0.1016 |
| *Acinetobacter baumannii* | 345 (6.7) | 139 (7.3) | 94 (5.7) | 112 (7.1) | 0.1512 |
| *Stenotrophomonas maltophilia* | 47 (0.9) | 21 (1.1) | 10 (0.6) | 16 (1.0) | 0.2670 |
| *Aeromonas hydrophila* | 20 (0.4) | 8 (0.4) | 11 (0.7) | 1 (0.1) | 0.0235 |
| Other | 57 (1.1) | 13 (0.7) | 14 (0.8) | 30 (1.9) | 0.0015 |
