# Supplementary figures and images for: Update of incidence and antimicrobial susceptibility trends of Escherichia coli and Klebsiella pneumoniae isolates from Chinese intra-abdominal infection patients
Source: BMC Infect Dis. 2017 Dec 18;17:776. doi: 10.1186/s12879-017-2873-z (PMC5735800; doi:10.1186/s12879-017-2873-z)

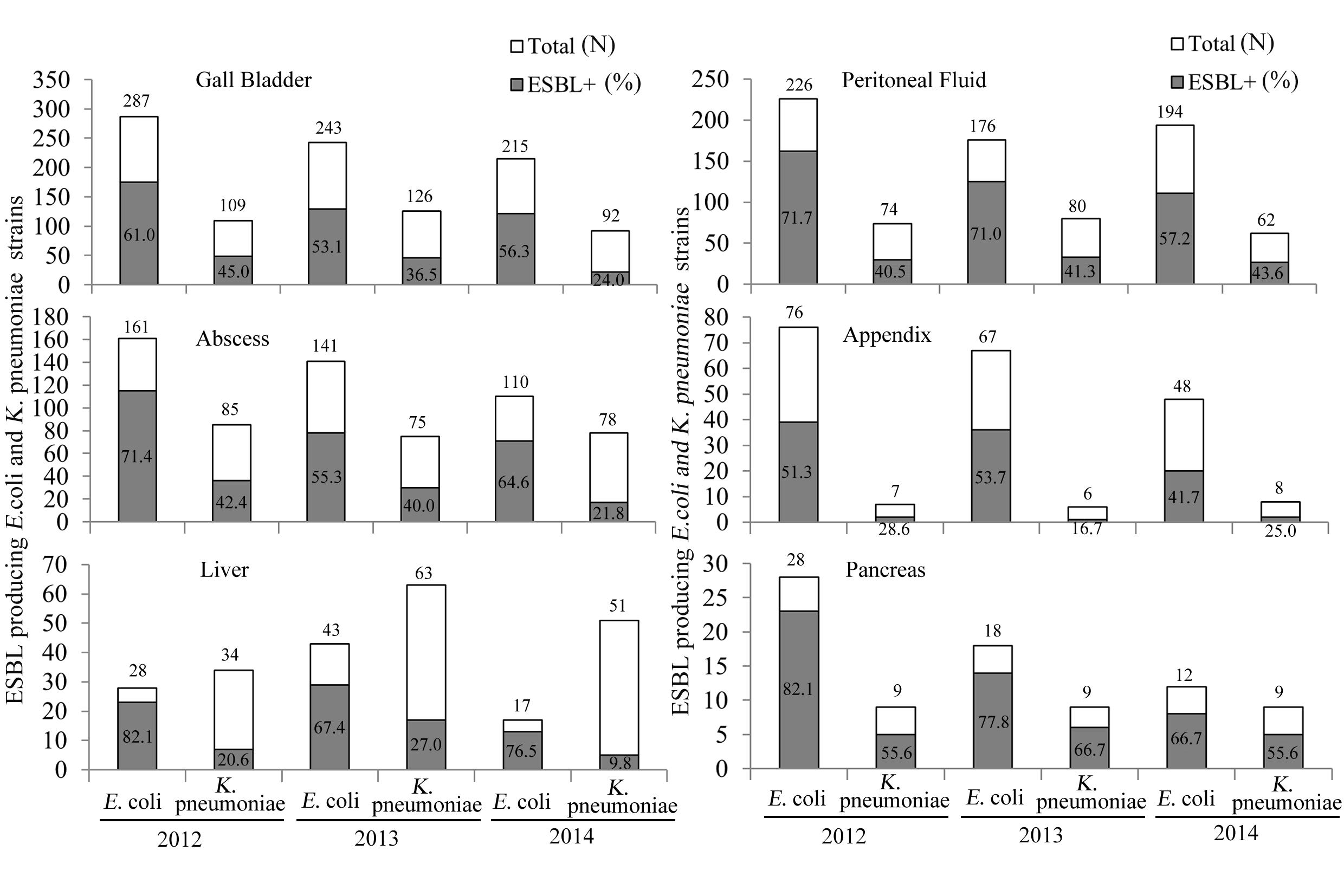

Supplement: Supplementary file 2 — Sources of ESBL-screen positive Escherichia coli and Klebsiella pneumoniae IAI isolates from 2012 to 2014. The upper numbers indicate the total number of isolates and the grey areas and numbers in the grey areas of the bars indicate the percentage of ESBL+ strains. (TIFF 748 kb) [file 12879_2017_2873_MOESM2_ESM.tif]
